# Modelling mass and heat transfer in nano-based cancer hyperthermia

**DOI:** 10.1098/rsos.150447

**Published:** 2015-10-21

**Authors:** M. Nabil, P. Decuzzi, P. Zunino

**Affiliations:** 1 Department of Mechanical Engineering and Materials Science, University of Pittsburgh, Pittsburgh, PA, USA; 2 Department of Translational Imaging, Houston Methodist Research Institute, Houston, TX, USA; 3 Modeling and Scientific Computing (MOX), Department of Mathematics, Politecnico di Milano, Milan, Italy

**Keywords:** nano-based cancer hyperthermia, microcirculation, mass and heat transfer, computer simulation

## Abstract

We derive a sophisticated mathematical model for coupled heat and mass transport in the tumour microenvironment and we apply it to study nanoparticle delivery and hyperthermic treatment of cancer. The model has the unique ability of combining the following features: (i) realistic vasculature; (ii) coupled capillary and interstitial flow; (iii) coupled capillary and interstitial mass transfer applied to nanoparticles; and (iv) coupled capillary and interstitial heat transfer, which are the fundamental mechanisms governing nano-based hyperthermic treatment. This is an improvement with respect to previous modelling approaches, where the effect of blood perfusion on heat transfer is modelled in a spatially averaged form. We analyse the time evolution and the spatial distribution of particles and temperature in a tumour mass treated with superparamagnetic nanoparticles excited by an alternating magnetic field. By means of numerical experiments, we synthesize scaling laws that illustrate how nano-based hyperthermia depends on tumour size and vascularity. In particular, we identify two distinct mechanisms that regulate the distribution of particle and temperature, which are characterized by perfusion and diffusion, respectively.

## 1. Introduction

Hyperthermia is a well-known method for cancer treatment, whereby the malignant tissue is subjected to high temperature to induce cell death, and eventually tumour shrinkage [1,2]. Hyperthermia has also been successfully used in combination with other methods of tumour treatment, e.g. chemotherapy and radiation [3–8], showing a considerable decrease in the size of tumour [3,8,9]. As an example, Coleman *et al.* [10] studied regional hyperthermia in which the tumour tissue has been heated to keep its temperature above 42°C for a few hours. However, among the concurrent treatments of cancer, hyperthermia seems to be the less developed in the clinical practice [11–13]. One reason may be the difficulty of targeting sufficient amounts of heat to the tumour tissue only. Another limitation is the intrinsic difficulty to tune the heat dose [14,15]. Indeed, the efficacy of hyperthermia treatment depends on several factors, including the maximum achieved temperature, the total time of heating and the tumour tissue properties [3,16]. Modern nanomedicine technology allows for targeted heat delivery by accumulating nanoparticles into a tumour mass and for accurately modulating the generated heat [17–23].

Over the past years, since early studies on whole-body hyperthermia to more recent applications targeting a confined tumour mass, mathematical and computational modelling have significantly contributed to understand the underlying physics of hyperthermia. For example, Jain [24] introduced simple distributed and lumped mathematical models to predict the temperature field during hyperthermia in normal and neoplastic mammalian tissues. Volpe & Jain [25] proposed and tested a 45-term lumped mathematical model to examine the average temperature distribution and thermal responses of the body under different clinical whole-body hyperthermia techniques. More recently, the efficiency of hyperthermia has been advanced by using nanoscale technologies. Specifically, gold-based nanoparticles (AuNPs), carbon-based nanoparticles (CNPs) and iron oxide nanoparticles (IONPs) appear to be the most promising nano-sized constructs for improving hyperthermia. AuNPs and CNPs can absorb near-infrared light, and therefore have been used in tumour photothermal therapy [26–32], whereas IONPs can generate heat by applying an alternating magnetic field (AMF) [33]. The latter method has two main advantages. Using IONPs, there is no limit in penetration depth into the tumour tissue. Also, clinical magnetic resonance imaging (MRI) can easily detect IONPs in human bodies [34–36]. Small nanoparticles with a diameter of less than 100 nm are necessary for application in biomedicine. If the magnetic core of IONPs is smaller than 20 nm, Brownian relaxation is the dominant mechanism for heat generation [33]. Specific absorption rate (SAR) is a key property of IONPs, which quantifies the efficacy of nanoparticles in generation of heat after exposure to the electromagnetic waves. A lot of recent *in vitro* studies have focused on characterizing SAR [35–41] and determining the corresponding tumour temperature. For example, von Maltzahn *et al.* [28] employed a transient three-dimensional finite-element heat transfer model as well as experimental measurements to investigate the photothermal tumour ablation by using gold nanorods. The results highlight the potential of numerical simulations coupled with the experiments as a new route for tumour therapy optimization and planning. Huang *et al.* [42] used a coupled axisymmetric three-dimensional cell death and heat transfer model in order to solve for the spatio-temporal distribution of injured cancerous cells and temperature field in human prostate. The predictions of the model successfully agreed with the performed experiments on different gold nanorods solutions heated by the near-infrared laser irradiation technique. This study arises from the results of Cervadoro *et al.* [17] where commercially available formulations of superparamagnetic iron oxide nanoparticles are thoroughly characterized in terms of SAR and absolute temperature increase.

The previous examples of nanoparticle-based hyperthermia show that this technology is the outcome of complex multiphysics interactions at the level of tumour microenvironment. The main effects that govern the process are: (i) blood perfusion; (ii) particle transport and interaction with tissue; and (iii) heat generation and transfer. Predictive models of these effects play a critical role to guide animal experiments and design better particle and heat delivery strategies. We aim to develop a sophisticated mathematical model, complemented by advanced computational techniques, that is able to accurately capture these phenomena. In particular, the proposed model has the unique ability of combining, for the first time, to the best of our knowledge, the following features: (i) realistic vasculature; (ii) coupled capillary and interstitial flow; (iii) coupled capillary and interstitial mass transfer applied to nanoparticles; and (iv) coupled capillary and interstitial heat transfer, which are the fundamental mechanisms governing nano-based hyperthermic treatment. This is an improvement with respect to previous modelling approaches, such that the Pennes’ bioheat equation [43], where the effect of blood perfusion on heat transfer is modelled in a spatially averaged form. In particular, our model accounts for capillary leakage, by means of a two-way coupling between the capillary network and the surrounding environment (i.e. the interstitial volume). By coupling blood perfusion with mass and heat transport equations in the vascular network and interstitial volume, we analyse the time evolution and the spatial distribution of particles and temperature in the targeted tumour.

We use the model to run virtual experiments of nano-based hyperthermia, which consist of direct numerical simulations of flow, mass and heat transport in the tumour microenvironment. The array of experiments is designed to elucidate how several quantities of interest for hyperthermia, such as the injected concentrations of nanoparticles, the particle accumulation and the temperature increase, scale with respect to tumour size. This phase of the study also represents a qualitative validation of the model based on the experiments presented in [17,44,45]. By means of a synergistic interaction of modelling and simulation, we synthesize and validate simple scaling laws that characterize how hyperthermia depends on tumour size. These laws may represent helpful guidelines to determine the adequate dosage of hyperthermia in clinical practice. Ultimately, the spirit of this work is aligned with the *precision medicine initiative* [46] for promoting quantitative approaches in support of more effective and personalized treatments of cancer, in particular, and other major diseases in general.

## 2. Models and methods

The mathematical model presented in this work is divided into three interacting modules:

(i) the capillary network coupled with interstitial filtration (presented in equation (2.1));
(ii) transport of particles (described by equation (2.2)); and
(iii) heat generation and transfer (modelled by equation (2.3))

**Figure 1.**
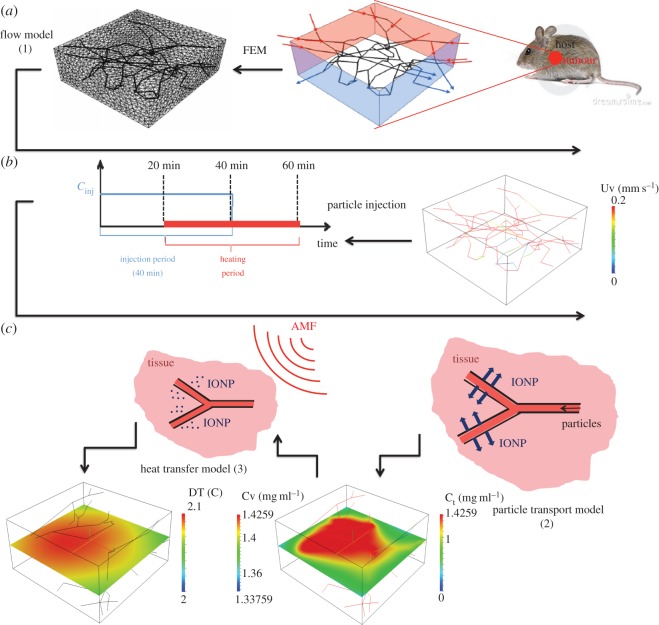
Computational approach subdivided into three main phases: (*a*) geometrical modelling of the tumour slab and generation of the computational meshes of *Ω* and *Λ*; (*b*) simulation of the blood, interstitial flow (only blood velocity field is visualized) and definition of the THT protocol; and (*c*) simulation of particle release (right panel) and heating upon irradiation of AMF (left panel).

As schematically described in figure 1, these are coupled phenomena, in the sense that each of them is affected by the previous one. As a result, they have to be solved in the order they are presented here. These phenomena can be modelled by means of space–time-dependent partial differential equations that can be efficiently solved using an advanced numerical technique called the *embedded multiscale method*. This computational approach has been developed in [47,48] and has been adapted here to a more general setting, encompassing heat transfer. For the sake of simplicity and clarity, each model is presented using the following schematic: *assumptions*; *notation and governing equations*; *boundary and initial conditions* and *constitutive laws and parameters*.

The geometrical model used in this study is shown in figure 1 and represents a tumour slab of R3230AC mammary carcinoma in rat dorsal skin flap preparation obtained in [49]. The dimensions of the slab are 550×520×230 10^−6^ m, and the capillary radius is *R*=7.64×10^−6^ m. Because the slab embeds a realistic microvascular geometry, it is decomposed into the capillary bed and the tumour interstitium, *Λ* and *Ω*, respectively. The variables defined on the capillary network will be labelled with the subscript *v* (*vascular*), whereas those of the tumour tissue are denoted with *t* (*tissue*). The model is made available, thanks to the *The Microcirculation Physiome Project* [50].

Our simulations describe a therapy protocol where the tumour slab is infused with a solution of particles. Particles reach the tumour slab through the inflow sections of the vascular network. These are identified by the intersections of the network with selected sides of the slab (highlighted in figure 1 as the light-red-coloured sides).

### 2.1 Flow

#### 2.1.1 Assumptions

The flow model consists of two parts, the microcirculation and the flow in the interstitial volume, which interact through suitable interface conditions modelling the capillary wall as a semipermeable membrane. It is assumed that the tumour interstitium behaves as an isotropic porous medium. The flow through the interstitium is modelled by the Darcy’s law of filtration. A Newtonian model is applied to the blood flow in the capillaries. Lymphatic drainage is also described, using the approach of [51,52]. Microcirculation is an extreme case where the size of vessels is the smallest, and the effect of blood pulsation is almost negligible. The Reynolds and the Womersley numbers characterizing the flow are very low when compared with other regions of the vascular network. As a result, Poiseuille’s law for laminar stationary flow of incompressible viscous fluid is adopted [53,54]. More precisely, we model each vascular branch as a one-dimensional channel. As shown in [54–57], this approximation significantly simplifies the problem at the computational level. We denote with *s* the arc length coordinate along each vascular branch and with **λ** the reference vector that identifies the orientation of each branch.

#### 2.1.2 Notation and governing equations

The coupled problem for microcirculation and interstitial flow consists to find the pressure fields *p*_t_, *p*_v_ and the velocity fields **u**_t_, **u**_v_ that satisfy the following equations,

2.1*a* $$\begin{array}{rlr} & -\nabla \cdot \left(\frac{\kappa }{\mu }\nabla p_{t}\right)+L_{p}^{LF}\frac{s}{v}(p_{t}-p_{L})-f_{b}(p_{t},p_{v})\delta_{\Lambda }=0 & \;\text{in}\;\Omega , \end{array}$$

2.1*b* $$\begin{array}{rlr} & {\text{u}}_{t}=-\frac{\kappa }{\mu }\nabla p_{t} & \;\text{in }\Omega , \end{array}$$

2.1*c* $$\begin{array}{rlr} & -\frac{\pi R^{4}}{8\mu }\frac{\partial^{2}p_{v}}{\partial s^{2}}+f_{b}(p_{t},p_{v})=0 & \;s\in \Lambda , \end{array}$$

2.1*d* $$\begin{array}{rlr} & f_{b}(p_{t},p_{v})=2\pi RL_{p}((p_{v}-p_{t})-\sigma^{p}(\pi_{v}^{p}-\pi_{t}^{p})) & \;\text{in}\;\Lambda , \end{array}$$

2.1*e* $$\begin{array}{rlr} & {\text{u}}_{v}=-\frac{R^{2}}{8\mu }\frac{\partial p_{v}}{\partial s}\lambda & s\in \Lambda \end{array}$$

2.1*f* $$\begin{array}{rlr} \;\text{and}\;\; & -\frac{\kappa }{\mu }\nabla p_{t}\cdot \text{n}=\beta_{b}(p_{t}-p_{0}) & \;\text{on}\;\partial \Omega . \end{array}$$

#### 2.1.3 Boundary conditions

A pressure drop along the capillary network is enforced. Because the inflow and outflow of the network are located on the lateral side of the tumour slab, a given pressure *p*_in_ is imposed on the two adjacent inlet faces, as indicated by a red colour in top left part of figure 1. Another pressure value *p*_out_ is set on the opposite sides. Using the Poiseuille’s law, the pressure drop *p*_in_−*p*_out_ is calculated in order to obtain a blood flow velocity equal to 0.2 mm s^−1^ in average which is a representative value for tumour blood flow according to Intaglietta *et al*. [58]. For the interstitial flow, Robin-type boundary conditions (2.1f) are imposed, where *p*_0_ denotes the pressure value at far field, whereas *β*_*b*_ represents an effective flow conductivity accounting for layers of tissue surrounding the tumour sample.

#### 2.1.4 Constitutive laws and parameters

Let *L*_p_ be the hydraulic permeability of the vessel wall (table 2 for units and physiological values) and let *p*_v_−*p*_t_ be the pressure difference between the vessels and the interstitial volume. Because of osmosis, the pressure drop across the capillary wall is affected by the difference in concentration of the chemicals dissolved in blood, [59,60], which determine the oncotic pressure jump $\left(\pi_{v}^{p}-\pi_{t}^{p}\right)$ modulated by the sieving coefficient *σ*^*p*^. In order to model the capillary phenotype typically observed in tumours, we increase the magnitude of their hydraulic permeability as in [51], such that the model will account of the well-known enhanced permeability and retention effect (EPR). To balance leakage of arterial capillaries, venous and the lymphatic systems absorb the fluid in excess. For the sake of generality, we include lymphatic drainage in the model, although the lymphatic system may be disfunctional in tumours. Following [51,52], we model them as a distributed sink term in the interstitial volume. It is assumed that the volumetric flow rate owing to lymphatic vessels, *Φ*^*LF*^, is proportional to the pressure difference between the interstitium and the lymphatics, namely $\Phi^{LF}(p_{t})=L_{p}^{LF}\left(s/v\right)(p_{t}-p_{L})$, where *L*^*LF*^_p_ is the hydraulic permeability of the lymphatic wall, *s*/*v* is the surface area of lymphatic vessels per unit volume of tissue and *p*_L_ is the hydrostatic pressure within the lymphatic channels. Because hydraulic permeability and lymphatics play an important role in determining the EPR effect in tumours, in §4.3, we analyse the sensitivity of computational simulations to these parameters.

### 2.2 Mass transport model

This model governs the distribution of magnetic material in the capillaries and the surrounding interstitial volume. Iron oxide is delivered by means of intravascular injection of IONP. We denote with *c*_v_ and *c*_t_ the iron oxide concentrations (mass/volume) in the vasculature and tissue, respectively.

#### 2.2.1 Assumptions

Particle transport in the capillary bed is modelled by means of advection–diffusion equations. Thanks to their small size, IONP can extravasate and diffuse in the interstitial tissue that is described as a homogeneous porous medium. Extravasation is governed by the assumption that capillary walls behave as semipermeable membranes.

#### 2.2.2 Notation and governing equations

Given blood flow and interstitial filtration **u**_v_,**u**_t_, respectively, the coupled problem accounting for transport of chemicals from the microvasculature to the interstitium consists to find the concentrations *c*_v_ and *c*_t_ such that,

2.2*a* $$\begin{array}{rlr} & \frac{\partial c_{v}}{\partial t}+\frac{\partial }{\partial s}\left(({\text{u}}_{v}\cdot \lambda )c_{v}-D_{v}\frac{\partial c_{v}}{\partial s}\right)=-\frac{1}{\pi R^{2}}f_{c}(p_{t},p_{v},c_{t},c_{v}) & \;\text{in}\;\Lambda \times (0,t), \end{array}$$

2.2*b* $$\begin{array}{rlr} & f_{c}(p_{t},p_{v},c_{t},c_{v})=2\pi R[L_{p}((p_{v}-p_{t})-\sigma^{p}(\pi_{v}^{p}-\pi_{t}^{p}))c_{v}+P(c_{v}-c_{t})] & \;\text{in}\;\Lambda , \end{array}$$

2.2*c* $$\begin{array}{rlr} & \frac{\partial c_{t}}{\partial t}+\nabla \cdot (c_{t}{\text{u}}_{t}-D_{t}\nabla c_{t})+L_{p}^{LF}\frac{s}{v}(p_{t}-p_{L})c_{t}=f_{c}(p_{t},p_{v},c_{t},c_{v})\delta_{\Lambda } & \;\text{in}\;\Omega \times (0,t), \end{array}$$

2.2*d* $$\begin{array}{rlr} & (c_{t}{\text{u}}_{t}-D_{t}\nabla c_{t})\cdot \text{n}=\beta_{c}c_{t} & \;\text{on}\;\partial \Omega \times (0,t) \end{array}$$

2.2*e* $$\begin{array}{rl} \;\text{and}\; & D_{v}=\frac{k_{B}T}{6\pi \mu r},\;D_{t}=D_{v}\frac{2(1-\epsilon )}{2+\epsilon }. \end{array}$$

In the post-processing phase, after the concentrations *c*_v_ and *c*_t_ have been determined, we calculate the average concentration of iron oxide in the tumour slab, defined as $c_{\mathrm{ref}}=|\Omega {|}^{-1}(\int_{\Omega }c_{t}+\int_{\Lambda }\pi R^{2}c_{v})$, which is one of the main factors that control tumour hyperthermia.

#### 2.2.3 Boundary and initial conditions

We posit that a constant concentration of iron oxide, denoted by *c*_inj_, is available in the blood flowing into the slab through the inflow sections of the vasculature. The particles are set free to leave the system though the complementary outflow boundaries. At the initial time, the vascular network and the tumour slab do not contain particles. For closing the transport problem of IONP, we model the layers of tissue surrounding the tumour sample by means of a condition that prescribes the flow resistance owing to the outer layers of tissue, namely equation (2.2d).

#### 2.2.4 Constitutive laws and parameters

IONP diffusivity *D*_v_ is estimated from the Stokes–Einstein relation while the value of *D*_t_ follows from the vascular diffusivity by means of the Maxwell mixture theory as in [61], see in particular equation (2.2e). These models are based on an idealized system of particles of radius *r* diffusing in a fluid of viscosity *μ* at room temperature *T* or into a saturated porous medium of volume fraction *ϵ*=0.8 (see also table 2).

### 2.3 Heat transfer model

#### 2.3.1 Assumptions

As extensively studied in [17], heat is a consequence of IONP irradiation with an AMF. This effect is modelled by means of a source term, *f*_*T*_(*c*_t_,*c*_v_), in the equation governing heat transfer in the tumour tissue. Because we simulate a short time scale and heat is generated in a small portion of the host body, we assume blood temperature homeostasis. As a result, blood temperature *T*_bl_ is constant in our model.

#### 2.3.2 Notation and governing equations

We study the temperature distribution (*T*) in the tumour. According to the previous assumptions, this variable is modelled by the following equations that encompass heat diffusion and convection by interstitial flow, heat absorption by lymphatic and capillary drainage and heat loss through the boundaries of the slab,

2.3*a* $$\begin{array}{rl} & \rho \gamma \left[\frac{\partial T}{\partial t}+\nabla \cdot \left(T{\text{u}}_{t}-\frac{\kappa }{\rho \gamma }\nabla T\right)+L_{p}^{LF}\frac{s}{v}(p_{t}-p_{L})(T-T_{\mathrm{bl}})\right]\\ & \;+2\pi R\beta_{T}(T-T_{\mathrm{bl}})\delta_{\Lambda }=\mathrm{SAR}(c_{t}+\pi R^{2}c_{v}\delta_{\Lambda })\;\text{in}\;\Omega \times (0,t) \end{array}$$

and

2.3*b* $$\;\left(-\frac{\kappa }{\rho \gamma }\nabla T+T{\text{u}}_{t}\right)\cdot \text{n}=\frac{\beta_{T}}{\rho \gamma }(T-T_{\mathrm{bl}})\;\text{on}\;\partial \Omega \times (0,t).$$

#### 2.3.3 Boundary and initial conditions

Robin-type boundary conditions are enforced for heat transfer across the outer boundaries of the tumour slab, see equation (2.3b), accounting for heat flux through tissue layers surrounding the tumour slab. At the initial time, the entire tumour tissue is set at the reference blood temperature *T*_bl_.

#### 2.3.4 Constitutive laws and parameters

Model parameters used for the simulations are reported in table 2. The thermophysical properties of the tissue, i.e. density (*ρ*), specific heat capacity (*γ*) and thermal conductivity (*κ*), all come from [17], as well as the values of blood temperature, size and SAR of magnetic nanoparticles. The coefficient *β*_*T*_ is the heat conductivity of the vascular walls, used to model how much heat is absorbed by the microcirculation. Its value comes from [62].

### 2.4 Computational solver

The discretization of problems (2.1)–(2.3) is performed by using the finite-element method. After partitioning into small elements the tumour and vasculature domains, *Ω* and *Λ*, respectively (figure 1 top right panel shows a representative computational domain of only 32 624 tetrahedral elements), the solutions of the governing equations are approximated with piecewise polynomial functions in the framework of the variational formulation. In particular, piecewise linear finite-elements are used for all the unknowns, namely *p*_t_,*c*_t_,*T*_t_ and *p*_v_,*c*_v_,*T*_v_, on a computational grid consisting of 49 655 grid points and 272 872 tetrahedral elements. Velocities **u**_t_,**u**_v_ are reconstructed in the post-processing phase using the pressure fields. We have adopted the generalized minimal residual method with incomplete-LU preconditioning to solve the algebraic systems following from the finite-element discretization. The sensitivity of the results with respect to the mesh size has been tested, and mesh independence was shown for grids finer than 257 109 elements.

We note that domains *Ω* and *Λ* feature heterogeneous dimensionality. The former is three-dimensional, the latter is one-dimensional. In order to model the natural leakage of capillaries, we apply the *embedded multiscale method* [47,48,63,64], which consists of representing the capillary bed as a network of one-dimensional channels acting as concentrated sources of flow immersed into the interstitial volume. The main advantage of the proposed scheme is that the computational grids required to approximate the equations on the capillary network and on the interstitial volume are completely independent. As a result, arbitrarily complex microvascular geometries can be studied with modest computational effort. From the standpoint of numerical approximation, the theoretical aspects of the method have been addressed in the works by D’Angelo [63,64]. These algorithms have been implemented using GetFem++, a general C++ finite-element library [65].

## 3. Results

We present here the results of a sequence of numerical experiments that we have performed to analyse tumour hyperthermic treatment (THT).

### 3.1 *In silico* testing protocol

Because it is a relatively unexplored treatment for cancer, a standard protocol for nano-based hyperthermia is not yet available. We have designed our virtual experiments using data from previous studies. For example, Johannsen *et al*. [66] studied clinical hyperthermia treatment on patients suffering from prostate cancer using iron oxide magnetic nanoparticles. The treatment plan included 60 min hyperthermia sessions following very slow injection of magnetic fluid. According to the review [67], which surveys the clinical applications of magnetic nanoparticles for both MRI and cancer hyperthermia treatment, iron oxide nanoparticles are usually injected by drip infusion method over 30 min. Furthermore, in [68], the experimental investigation of plasmonic silica/gold nanoshells applied to tumour photothermal treatment of rats was reported. Every animal was subjected to two separate 20 min period of injection, i.e. one subcutaneously and the other one intramuscularly.

On the basis of these examples, we analyse a time interval of 60 min where for the initial 40 min the tumour is supplied with a solution of IONP, as a consequence of intravascular injection of particles into the host. The underlying assumption is that, for a small animal, the intravenous infusion of magnetic fluid directly affects the blood concentration in the entire systemic circulation, which we denote as *c*_inj_. In the interval 20–60 min, the tumour is exposed to a low-frequency AMF of 500 kHz that excites the particles and generates heat according to the prescribed specific heat absorption parameter (SAR=10^6^ W kg^−1^) for IONP. As shown in [17], low-frequency AMF is desirable because it does not generate non-specific heat owing to excitation of the electrolytes dissolved in the interstitial fluid.

We recall that the average concentration of iron oxide in the tumour slab, denoted as *c*_ref_, previously introduced together with systems (2.2), is one of the main factors that control THT in the tumour slab. In particular, we have chosen to run experiments targeting the reference value *c*_ref_=1 mg ml^−1^ because it matches the injected concentrations used in the experiments of Cervadoro *et al*. [17].

### 3.2 Sensitivity and scaling analysis at equilibrium

Using mass and energy balance analysis, we derive approximate formulae for the dependence of the temperature increase relative to blood basal temperature, namely Δ*T*=*T*−*T*_bl_, on the model parameters and the size of the tumour slab. Here, time and space dependence are neglected. This approach has the main advantage to end up with algebraic equations that can be easily solved. Although the resulting equations are significantly less accurate than (2.1)–(2.3), they are extremely helpful to gain better insights into the main mechanisms that determine particle distribution and temperature increase. The derivation of the simplified model relies on the following assumptions:

(i) *transport and thermal equilibrium*: for any model variable *v*, we have *v*(*t*,**x**)=*v*(**x**) and
(ii) *uniform concentration and temperature fields*: it entails that *v*(**x**)=*v* is a constant.

Let us consider equation (2.3a) and integrate it over *Ω*. According to assumption (i), we drop the time derivative of the temperature, and we apply the divergence theorem to the second term on the left-hand side:

$$\begin{array}{rl} & \int_{\Omega }\rho \gamma \nabla \cdot \left(T{\text{u}}_{t}-\frac{\kappa }{\rho \gamma }\nabla T\right)d\text{x}+\int_{\Omega }\left(\rho \gamma L_{p}^{LF}\frac{s}{v}(p_{t}-p_{L})(T-T_{\mathrm{bl}})+2\pi R\beta_{T}(T-T_{\mathrm{bl}})\delta_{\Lambda }\right)d\text{x}\\ & \;=\int_{\partial \Omega }\beta_{T}(T-T_{\mathrm{bl}})\;d\text{s}+\int_{\Omega }\left(\rho \gamma L_{p}^{LF}\frac{s}{v}(p_{t}-p_{L})(T-T_{\mathrm{bl}})+\int_{\Lambda }2\pi R\beta_{T}(T-T_{\mathrm{bl}})\right)d\text{x}\\ & \;=\int_{\Omega }f_{T}(c_{t},c_{v})\;d\text{x}. \end{array}$$

Then, owing to assumption (ii), the previous equation leads to

3.1 $$\Delta T\left(\beta_{T}|\partial \Omega |+\rho \gamma L_{p}^{LF}\frac{s}{v}(p_{t}-p_{L})|\Omega |+2\pi R\beta_{T}|\Lambda |\right)=\mathrm{SAR}(c_{t}|\Omega |+\pi R^{2}c_{v}|\Lambda |),$$

where |*Ω*|, |∂*Ω*|, |*Λ*| denote the volume of *Ω*, its outer surface and the length of *Λ* respectively. Equation (3.1) allows us to determine the temperature increase as a result of simple calculations given the (average) particle concentrations *c*_t_,*c*_v_. Proceeding in a similar way for particle concentration, using in particular equation (2.2c), we obtain the following formula for the relation between vascular and tissue concentrations,

3.2 $$c_{t}=\frac{2\pi R[L_{p}((p_{v}-p_{t})-\sigma^{p}(\pi_{v}^{p}-\pi_{t}^{p}))+P]|\Lambda |}{\beta_{c}|\partial \Omega |+L_{p}^{LF}\left(s/v\right)(p_{t}-p_{L})|\Omega |+2\pi RP|\Lambda |}c_{v}.$$

We use models (3.1) and (3.2) to study how the hyperthermic treatment depends on the tumour size and other parameters, such as the *vascularity* (defined below). Let *Ω* be the image of a reference domain *Ω*^′^ obtained by a uniform scaling of the reference axes of a factor *δ*, namely **x**=*δ***x**^′^. As a result, we have

3.3 $$|\Omega |=\delta^{3}|\Omega^{\prime }|,\;|\partial \Omega |=\delta^{2}|\partial \Omega^{\prime }|,\;|\Lambda |=\delta |\Lambda^{\prime }|.$$

By changing the scaling factor *δ*, but not scaling the capillary radius proportionally, we modify the vascularity of the system that we define as follows:

3.4 $$\text{volumetric vascularity}\;=\frac{\text{volume of capillaries}}{\text{volume of slab}}=\frac{\pi R^{2}|\Lambda |}{|\Omega |}=\frac{\pi R^{2}|\Lambda^{\prime }|}{|\Omega^{\prime }|}\frac{1}{\delta^{2}}.$$

Finally, substituting definitions (3.3) into (3.1) and (3.2), we obtain the following approximate expressions for the effect of tumour size on temperature increase:

3.5*a* $$\begin{array}{rl} c_{t}(\delta ) & =c_{v}(\delta )\frac{2\pi R[L_{p}((p_{v}-p_{t})-\sigma^{p}(\pi_{v}^{p}-\pi_{t}^{p}))+P]|\Lambda^{\prime }|\delta }{\beta_{c}|\partial \Omega^{\prime }|\delta^{2}+L_{p}^{LF}\left(s/v\right)(p_{t}-p_{L})|\Omega^{\prime }|\delta^{3}+2\pi RP|\Lambda^{\prime }|\delta }, \end{array}$$

3.5*b* $$\begin{array}{rl} c_{\mathrm{ref}}(\delta ) & =c_{t}(\delta )+\frac{\pi R^{2}|\Lambda^{\prime }|}{|\Omega^{\prime }|}\frac{1}{\delta^{2}}c_{v}(\delta ) \end{array}$$

3.5*c* $$\begin{array}{rl} \;\text{and}\;\;\Delta T(\delta ) & =\frac{\mathrm{SAR}(|\Omega^{\prime }|\delta^{3}c_{t}(\delta )+\pi R^{2}|\Lambda^{\prime }|\delta c_{v}(\delta ))}{\left(\beta_{T}|\partial \Omega^{\prime }|\delta^{2}+\rho \gamma L_{p}^{LF}\left(s/v\right)(p_{t}-p_{L})|\Omega^{\prime }|\delta^{3}+2\pi R\beta_{T}|\Lambda^{\prime }|\delta \right)}. \end{array}$$

### 3.3 Description of the results

We use the computational model to perform the following studies.

**Figure 2.**
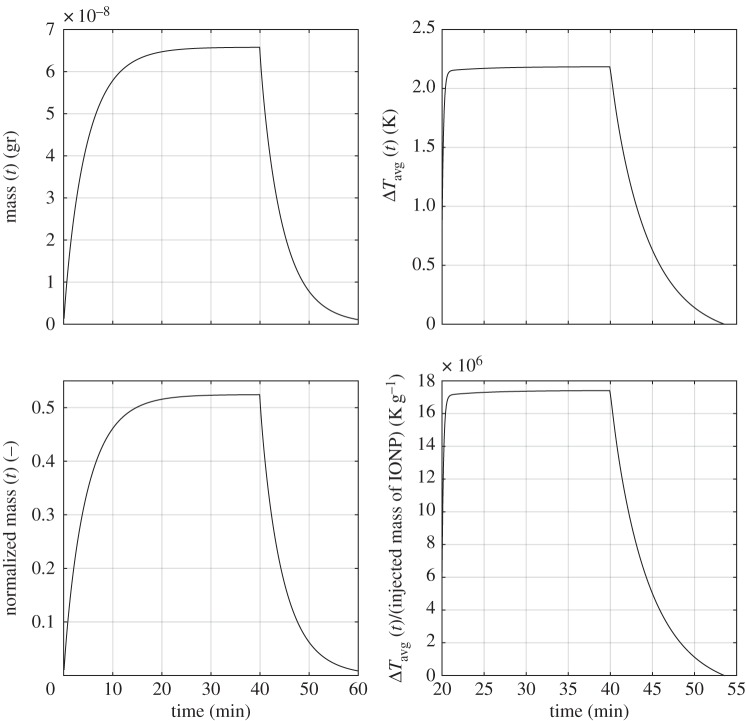
(Top panel) Timecourses of IONP accumulation (left) and temperature increase (right). (Bottom panel) The same dataset is shown after renormalization with respect to the amount of IONP injected for 40 min.

**Figure 3.**
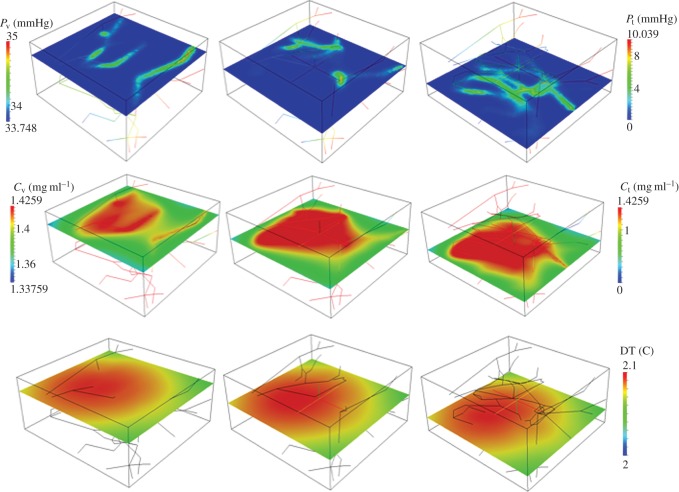
Interstitial fluid pressure, *p*_t_ for high hydraulic permeability, as in table 2, and active lymphatic drainage (top panels). Spatial distribution of *c*_t_,*c*_v_ for IONP delivery with constant *c*_ref_=1 mg ml^−1^ (middle panels) and corresponding temperature maps (bottom panels) after 40 min of particle injection and 20 min of AMF exposure.

**Figure 4.**
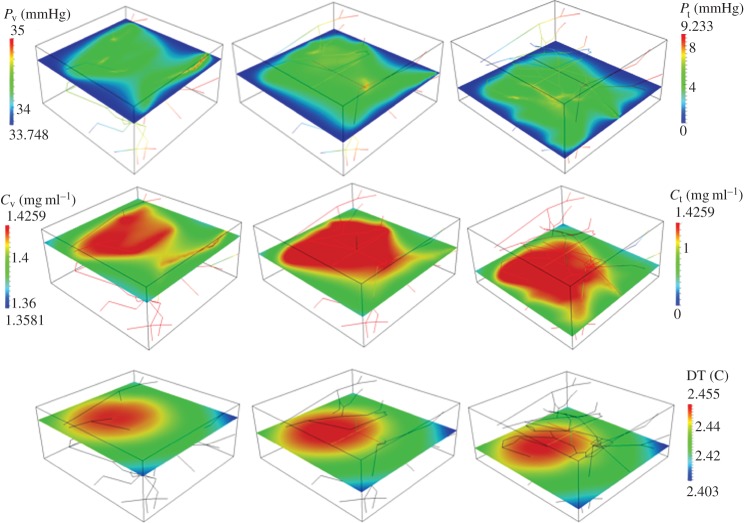
Interstitial fluid pressure, *p*_t_ for high hydraulic permeability and absent lymphatic drainage *L*^*LF*^_p_(*s*/*v*)=0 (top panels). Spatial distribution of *c*_t_,*c*_v_ for IONP delivery with constant *c*_ref_=1 mg ml^−1^ (middle panels) and corresponding temperature maps (bottom panels) after 40 min of particle injection and 20 min of AMF exposure.

**Figure 5.**
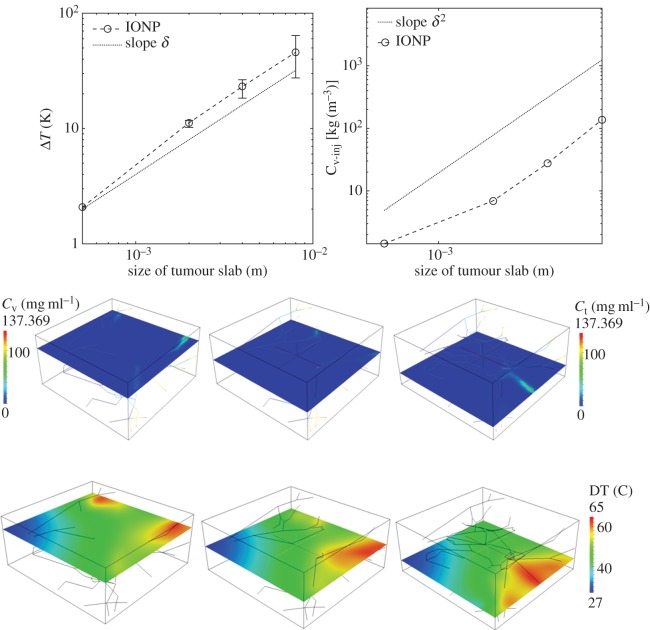
(Top panels) Hyperthermia predictions for variable tumour size (open circle, marker) when *c*_ref_ is fixed to 1 mg ml^−1^. The trend of the injected concentration of particles required to maintain *c*_ref_ constant is shown on the right. Error bars visualize the temperature heterogeneity, namely the difference $\max_{\Omega }\Delta T-\min_{\Omega }\Delta T$, when the tumour size increases. (Bottom panels) Calculated IONP concentration and temperature maps at time 40 min for tumour size of 8 mm (*δ*=16).

**Figure 6.**
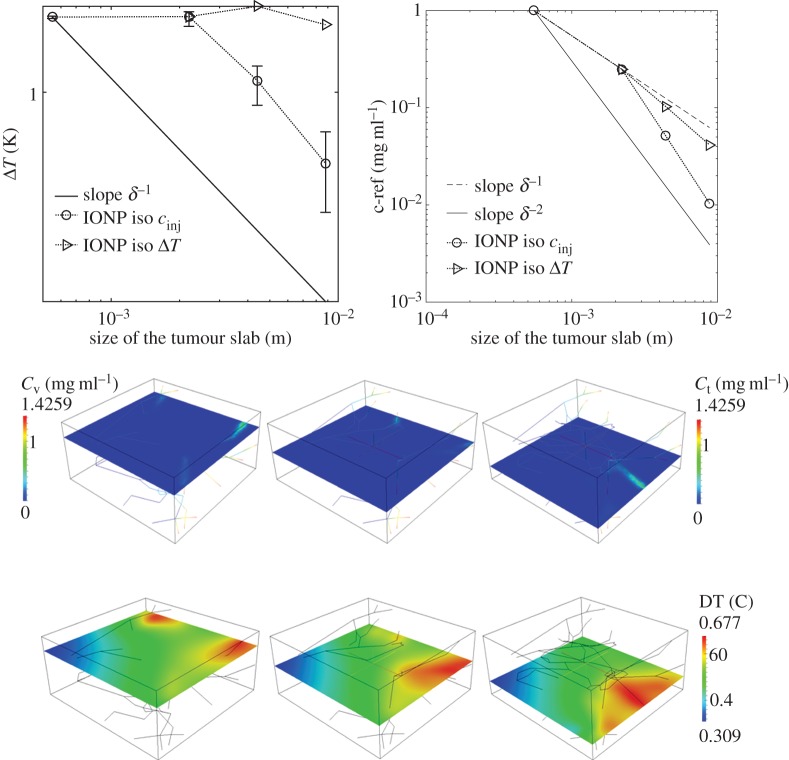
(Top panels) Hyperthermia predictions for variable tumour size for a fixed *c*_inj_=1.425 mg mm^−3^. Error bars quantify temperature heterogeneity. On the right, we show how the average IONP concentration in the tumour slab, *c*_ref_, decreases when the tumour size increases. Particle concentrations and temperature maps at 40 min for *δ*=16 are shown below.

**Figure 7.**
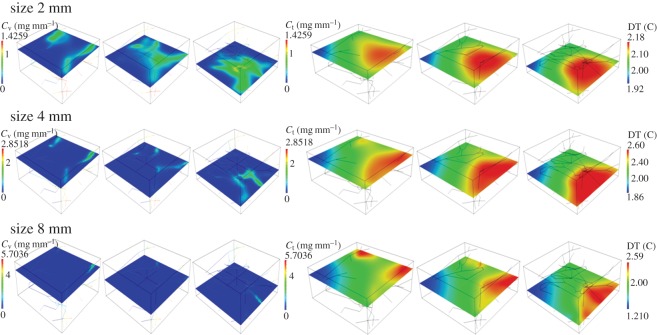
Temperature maps corresponding to the constant temperature dataset (marker Δ) of figure 6 (top left panel).

(i) Analysis of average particle concentration and average temperature timecourses during injection and heating. These results are reported in figure 2. Temperature evolution is only shown after 20 min, because AMF exposure goes from 20<*t*<40 min.
(ii) Combined spatial maps of interstitial fluid pressure (IFP), concentration and temperature. In particular, figure 3 shows particle concentration and temperature fields at 40 min, the time when particle injection is switched off. The role of lymphatic drainage on interstitial fluid pressure is analysed in figure 4, which differs from figure 3 because it addresses the case of disfunctional lymphatics.
(iii) Scaling analysis with respect to tumour size and vascularity for constant *c*_ref_. Referring to equation (3.3), we set as *Ω*^′^ the original tumour configuration of size 550×520×230 10^−6^ m and we set *δ*=4,8,16 for the numerical experiments on larger tumour sizes. Our simulation will span the typical tumour size used in animal experiments with mice models, such as in [17,44,45]. We note that the geometrical configuration of the vasculature remains unchanged. As a consequence, according to equation (3.4), the vascularity of the tumour decreases as *δ*^−2^ when the tumour size increases. Moreover, as far as we progressively increase the tumour size, we suitably modify the injected concentration (by increasing it as shown in figure 5 top right panel) in order to keep the reference concentration constant to *c*_ref_=1 mg ml. The results of numerical simulations are summarized in figure 5.
(iv) Scaling analysis with respect to tumour size and vascularity for constant *c*_inj_. In figure 6, the trends of average particle concentrations and tumour temperature are shown with respect to the tumour characteristic size together with visualizations of temperature and concentration maps equivalent to the ones of figure 5, for comparison.
(v) Sensitivity analysis of particle concentration and temperature increase when parameters such as the hydraulic permeability of the capillary wall *L*_p_, the permeability to particle extravasation *P*, and the lymphatic drainage coefficient *L*^*LF*^_p_(*s*/*v*) are varied through several orders of magnitude. The results, reported in figure 7, illustrate how the main findings of this work apply to a range of parameters wider than those reported in table 2.

## 4. Discussion

The extended spectrum of numerical experiments that we have performed allow us to elucidate the main characteristic traits of THT, also guided by the simplified model for temperature increase at equilibrium (3.5).

### 4.1 Analysis of iron oxide nanoparticles timecourses and temperature maps

In figure 2, the total particle mass delivered to the tumour slab and the corresponding average temperature increase are analysed. In these simulations, the injected particle density has been set to match reference slab concentration of 1 mg ml^−1^. Two characteristic traits of IONP delivery emerge. Figure 2 (bottom panels) shows the delivery efficiency, namely the mass of delivered particles and average temperature increase per unit mass of delivered material. More precisely, we plot

4.1 $$\frac{\int_{\Omega }c_{t}\;dx+\int_{\Lambda }\pi R^{2}c_{v}\;ds}{\int_{0}^{T}(\pi R^{2}c_{\mathrm{inj}}{\text{u}}_{\mathrm{in}}\cdot \lambda )\;dt}.$$

We observe that more than 50% of the injected particles are absorbed by the tumour slab, because of their ability to extravasate and diffuse within the interstitial volume. However, for similar reasons, small particles suffer from small residence times, as demonstrated by the quick drop of particle concentration and temperature after 40 min. In other words, IONP particle concentration significantly drops as soon as particle injection is switched off. These data suggest that IONP particle delivery and host exposure to AMF field should be synchronous in order to achieve an effective THT. The significant washout rate is one of the major disadvantages of injecting small particles. Although not yet validated by animal experiments, this theoretical conjecture is compatible with the general principles of nanomedicine for tumour treatment, in the sense that it is commonly expected that small particles, such as iron oxide nanocrystals, are subject to a more significant washout than larger constructs, such as particle clusters.

For the same simulations, particle concentration and temperature fields are shown in figure 3. The temperature increase, about 2 K, is consistent with the experiments of [17,45] and also in line with [44]. More precisely, in [17] (figure 6), a temperature increase of 5 K is predicted using a simpler version of the mathematical model addressed here, complemented with equivalent data on tumour average particle concentration and SAR. In [45], hyperthermia of about 4 K is observed in animals treated with a similar protocol (but a direct comparison on injected concentration of IONP is not available). For a highly vascularized small tumour (about 0.5 mm width), the particle distribution among the tissue is rather uniform. For this reason, the spatial variation (or spatial heterogeneity) of temperature is almost negligible. The conditions of this numerical experiment are ideal to assess the accuracy of the simplified model (3.1), which is based on equilibrium assumptions. Indeed, using the same parameters of the simulations, (3.1) provides an estimate of temperature increase, Δ*T*, of 2.3 K, which closely matches with the temperature data of figure 2. From equation (3.1), we observe that Δ*T* at equilibrium is determined by the balance of heat sources (right-hand side) and heat sinks (left-hand side). The latter consist of heat loss through boundaries, lymphatic and vascular heat absorption. Model (3.1) shows that the heat loss at tumour boundaries, i.e. *β*_*T*_|∂*Ω*| dominates over the other heat loss factors.

### 4.2 Analysis of tumour hyperthermic treatment sensitivity to tumour size

Figures 5 and 6 inform us about the sensitivity of THT with respect to tumour size for constant *c*_ref_ and *c*_inj_, respectively. In all cases, the sensitivity is high, both for particle concentrations and temperature increase. We note that when increasing the tumour size, proportionally to the parameter *δ* as in equation (3.3), we do not vary the capillary radius. As a result of that, the vascularity of the tumour mass depends on *δ* as shown in equation (3.4).

#### 4.2.1 Numerical simulations varying *δ* with constant *c*_ref_

To maintain constant particle concentration levels as long as tumour size increases (governed by the scaling parameter *δ*), the injected concentration of particles increases as *δ*^2^, quickly leading to injected concentration that can hardly be obtained in practice (see figure 5, top right panel). The interpretation of this scaling law is that in order to keep mass constant, the vascular concentration must compensate the decrease of volumetric vascularity, which scales as *δ*^−2^ according to equation (3.4). As a result, we have *c*_v_(*δ*)≃*δ*^2^. This behaviour is summarized in table 1. At equilibrium conditions, the value of *c*_ref_ is governed by equation (3.5b). Substituting into this equation *c*_ref_≃1 and *c*_v_(*δ*)≃*δ*^2^, we infer that *c*_t_≃1. As a result, *c*_v_(*δ*) dominates over *c*_t_(*δ*). In other words, blood particle concentration is the leading term that determines the total particle mass inside the tumour slab. This regime of particle delivery can be defined as *prefusion-dominated*.

**Table 1.** Scaling of the particle concentrations and THT with respect to tumour size.

	perfusion regime			diffusion regime
	*δ*>4			*δ*<4
vascularity	*δ*^−2^	*δ*^−2^	*δ*^−2^	*δ*^−2^
*c*_ref_	1	*δ*^−2^	*δ*^−1^	*δ*^−1^
*c*_inj_	*δ*^−2^	1	*δ*	1
Δ*T*	*δ*	*δ*^−1^	1	1
	figure 5	figure 6	figure 7	figure 6

As a consequence of these high levels of injected particles, Δ*T* increases with the tumour size, quickly reaching intolerable values (figure 5, top left panel). At equilibrium, temperature increase responds to the simple model (3.5c). The data on vascular concentration growth suggest that heating is dominated by *πR*^2^|*Λ*^′^|*δc*_v_(*δ*). Then, the numerator of (3.5c) scales as *δ*^3^, whereas the denominator scales as *δ*^2^, because the first term dominates over the others. As a consequence, injecting particles keeping constant concentration in the tumour slab results into a *linear* increase of Δ*T*. Increasing *δ* also augments temperature heterogeneity (visualized in figure 5, top left panel by means of error bars quantifying the gap between $\max_{\Omega }\Delta T$ and $\min_{\Omega }\Delta T$). For large tumours, the analysis of particle distribution patterns displayed in figure 5 (bottom panels) suggests that most of the IONP particles are delivered in the proximal part of the tumour mass, whereas the distal part of the vasculature carries low particle concentration because of particle sequestration upstream. This effect is the reason of the considerable variations of temperature increase. To avoid undesired temperature heterogeneity during THT, tumours should be treated in order to generate smooth neovasculature. This observation suggests that nano-based hyperthermia should be combined with vascular normalization [69].

#### 4.2.2 Numerical simulations varying *δ* with constant *c*_inj_

The simulation of injecting particles at constant *c*_inj_ for variable tumour size, *δ*=4,8,16, is addressed in figure 6. These results show that *c*_ref_≃*δ*^−2^. Equation (3.5b) confirms that for a constant *c*_inj_=1.425 mg mm^−3^, *c*_t_ and *c*_ref_ scale as *c*_t_(*δ*)≃*c*_ref_(*δ*)≃*δ*^−2^. This means that, as far as the tumour size increases, the blood particle concentration is the major source of heat, whereas the tissue concentration does not significantly contribute to THT. Even though the scaling laws are different from those of the case *c*_ref_=1, this delivery strategy can be defined as *perfusion-dominated*.

For a tumour size large enough, namely *δ*>4, the trend of temperature increase with size is visualized in figure 6 (top left panel) and it scales as Δ*T*≃*δ*^−1^. This trend can be explained using equation (3.5c), where the numerator scales as *δ* while the denominator is proportional to *δ*^2^, from which the observed behaviour Δ*T*≃*δ*^−1^ emerges. The analysis of concentration and temperature spatial maps shows a significant temperature heterogeneity, because particles are unevenly distributed. Temperature gradients correlate well with the orientation of vascular flow. In other words, the peripheral region of the tumour is more heated than the internal part.

From the previous results, we observe that neither the delivery strategy with constant tumour reference concentration, nor the one with constant injection concentration can achieve the desirable result of maintaining a constant THT when the tumour size varies above *δ*=4. Then, a natural question arises *what delivery strategy would maintain constant THT performance for different tumour sizes?* Thanks to the summary of mechanisms reported in table 1, the answer appears very naturally. The therapy guaranteeing that temperature increase Δ*T* is insensitive to tumour size lays halfway between the treatment with constant *c*_ref_ and the one with constant injection. More precisely, we posit that Δ*T* will remain constant by scaling *c*_inj_ linearly with respect to the tumour size. This assumption is indeed verified by the simulations reported in figure 7. The analysis of spatial distribution of temperature shows again that tumour periphery is overheated and tumour core is underheated.

The situation of IONP delivery in the case of small tumours, namely the interval *δ*<4 obeys mechanisms different from those illustrated so far. From figure 6 (top right panel), we observe that *c*_ref_≃*δ*^−1^ for constant particle injection. Recalling that *c*_inj_ is constant and that *β*_*c*_|∂*Ω*^′^|*δ*^2^ dominates over the other terms at the denominator, equation (3.5a) implies that *c*_t_≃*δ*^−1^. Then, substituting the asymptotic orders of *c*_t_ and *c*_v_ into (3.5b), we conclude that the concentration of particles in the interstitial space *c*_t_(*δ*) is the dominant component of *c*_ref_. We say that, in this case, particle and heat delivery is *diffusion-dominated*.

Equation (3.5c) entails that Δ*T* should be rather insensitive to tumour size, as confirmed by the simulations performed with the full model, (2.1)–(2.3). A comparison of the release and heating mechanisms for small and large tumours is provided in table 1. The diffusion-dominated regime may certainly enhance particle distribution within the tumour, with the desirable outcome of decoupling temperature increase from the effect of tumour size, but at the same time excessive leakage of particles into the whole body may reduce their accumulation in the tumour region.

### 4.3 Sensitivity analysis of capillary permeability and lymphatic drainage.

In figure 4, we analyse the effect of lymphatic drainage on IFP, particle distribution and temperature increase. In particular, the comparison of figures 3 and 4 informs about the sensitivity of these results on the functionality of the lymphatic system, which may be absent or impaired in tumours. Figures 3 and 4 show that lymphatic drainage has a significant effect on IFP. Indeed, high IFP occurs only in the neighbourhood of capillaries when drainage is active (figure 3), while it extends over the entire domain in the case of a dysfunctional lymphatic system (figure 4). However, particle distribution and hyperthermia are almost insensitive to changes in lymphatic drainage and IFP. This effect can be explained recalling that we are considering very small particles (significantly smaller than the nanoconstructs typically used for tumour therapy), whose transport across the tumour mass is diffusion-dominated. This analysis suggests that the general results of this work and in particular the scaling laws of table 1 remain valid in the case of absent or impaired lymphatic drainage in the tumour.

**Figure 8.**
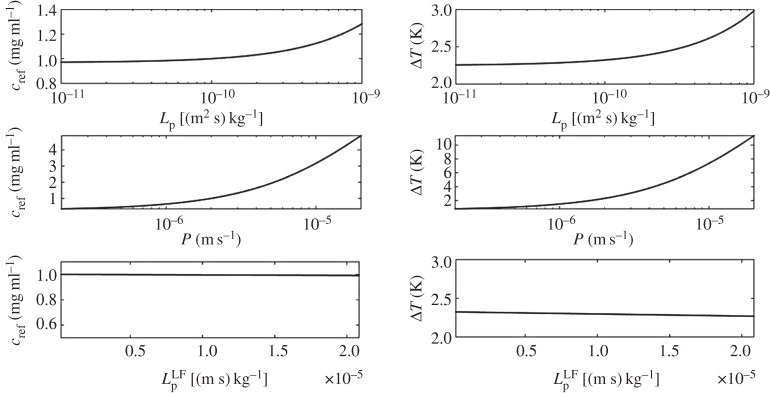
Sensitivity analysis of *c*_ref_ and Δ*T* with respect to variations of *L*_p_, *P* and *L*^*LF*^_p_ from top to bottom.

Using the expressions (3.5), derived under restrictive assumptions and validated using the full model, we study the sensitivity of average particle accumulation, *c*_ref_ and average temperature increase Δ*T*, when capillary permeability and lymphatic drainage depart from the values reported in table 2. The results of figure 8 show that the effect of *L*_p_ and *L*^*LF*^_p_ on the average particle concentration and temperature increase is not significant. Because the hydraulic and lymphatic permeabilities control the fluid extravasation and resorption, namely the EPR effect, we conclude that our results are quite insensitive to perturbations of EPR. The vascular permeability to particles, namely *P*, plays a more important role. This behaviour can be justified observing that we are considering very small particles that distribute into the tumour mass by means of a diffusion process.

**Table 2.** Parameters and data for mass and thermal transport.

symbol	parameter	units	value	source	equation
*L*_p_	hydraulic permeability, capillary wall	(m^2^ s) kg ^−1^	10^−10^	[47]	(2.2*b*)
*L*^*LF*^_p_(*s*/*v*)	effective permeability, lymphatic vessels	(mmHg h)^−1^	0.5	[47]	(2.2*c*)
*c*_inj_	inflow IONP concentration	gr m^−3^	1425.9	[47]	—
*d*	edge length of IONP	m	1×10^−8^	[17]	—
*m*	mass of IONP	gr	8×10^−18^	[17]	—
*D*_v_	vascular diffusivity of IONPs	m^2^ s^−1^	9.0687×10^−11^	[47]	(2.2*e*)
*D*_t_	interstitial diffusivity of IONPs	m^2^ s^−1^	1.2955×10^−11^	[47]	(2.2*e*)
*P*	vascular permeability of IONPs	m s^−1^	2×10^−6^	[47]	(2.2*b*)
*ρ*	tissue density	kg mm^−3^	1060×10^−9^	[17]	(2.3*a*)
*γ*	tissue-specific heat capacity	J kg^−1^ K^−1^	3470	[17]	(2.3*a*)
*κ*	tissue thermal conductivity	kg mm^−1^ K^−1^	0.51×10^−3^	[17]	(2.3*a*)
*β*_*T*_	heat exchange coefficient	W mm^−2^ K^−1^	2×10^−5^	[17]	(2.3*b*)
*T*_bl_	blood temperature	K	273.15+37	[17]	(2.3*a*)
SAR	specific absorption rate of IONPs	W kg^−1^	1×10^6^	[17]	(2.3*b*)
*ϵ*	tumour tissue volume fraction	—	0.8	[61]	(2.2*e*)

## 5. Conclusion

We have derived a novel mathematical model for coupled heat and mass transport in the tumour microenvironment and applied it to study nanoparticle delivery and hyperthermic treatment. Simulations show that capillary configuration and blood flow affect the distribution of delivered particles and the corresponding temperature field. Although a direct comparison would be required to fully assess the merits of the present approach, this observation suggests that our model is more adequate for studying nano-based hyperthermic treatment than those based on spatial averaging of the vascular network, such as the Pennes’ bioheat equation.

We have used the model, combined with state of art numerical methods, to analyse the effect of tumour size and vascularity on THT. By means of an array of numerical experiments, we have synthesized scaling laws that illustrate how hyperthermia depends on these parameters. In particular, we have identified two distinct regimes that regulate nano-based hyperthermia using IONP: the *perfusion* and the *diffusion*-dominated ones.

Ongoing work is oriented to studying the effect of particle size and vascular network configuration on hyperthermia. Besides providing new insights into the mechanisms governing nano-based hyperthermia, our methodology might facilitate the design of better clinical protocols for hyperthermia, in line with the *precision medicine initiative* [46] for innovative prevention and treatment strategies, focusing in particular on cancer.
